# Trust-building interventions to home-dwelling persons with dementia who resist care

**DOI:** 10.1177/09697330211041745

**Published:** 2022-02-22

**Authors:** Åshild Gjellestad, Trine Oksholm, Herdis Alvsvåg, Frøydis Bruvik

**Affiliations:** VID Specialized University, Norway; University of Bergen, Norway

**Keywords:** Care of the older person, dementia, ethics and dementia, ethics of care, home care, home healthcare, qualitative research, resistance to care, thematic analysis, trust-building interventions

## Abstract

**Background:**

Providing care for a home-dwelling person with dementia who resists care is an ethical and practical complex and challenging task. Faced with a growing number of persons with dementia, the healthcare professional’s understanding of how to best care for and prevent unnecessary use of coercion with persons with dementia is of key importance.

**Research aim:**

The aim of this study was to explore the use of trust-building interventions in home-dwelling persons with dementia resisting care, as described by health professionals in documents of decisions of forced treatment and care.

**Research design:**

A qualitative thematic document analysis inspired by critical realism was conducted.

**Participants and research context:**

Descriptions of trust-building interventions were extracted from 88 documents of forced treatment and care for home-dwelling persons with dementia, receiving home healthcare, in 2015 and 2016.

**Ethical considerations:**

Approved by the Regional Committee for Medical and Health Research Ethics, reference number 2017/788, and controlled by the Norwegian Centre for Research Data, reference number 54897. The study adhered to the guidelines of the Declaration of Helsinki.

**Findings:**

We found that “balancing safe care with the person’s integrity” was an overarching theme that permeated the descriptions of trust-building interventions in the study. Three main themes were identified when the data were analyzed: *safeguarding care*, *protecting integrity*, and *optimizing the environment.*

**Discussion and conclusion:**

Health professionals balanced on a thin line between care and integrity when met with resistance from person with dementia. However, the trust-building interventions used in the most challenging situations did not differ from the interventions used in general in dementia care. Two knowledge gaps were identified: how to perform appropriate assessments of situations of home-dwelling persons with dementia when met with resistance to care, and whether environmental initiatives may also benefit home-dwelling persons with dementia who are not easily cared for.

## Introduction

Providing healthcare services for a home-dwelling person with dementia (PWD) who resists care is an ethical and practical complex and challenging task.^1–3^ Resistance to care due to dementia increases the risk of using forced treatment and care.^4^ Person-centered approaches are one way to prevent or reduce the use of forced treatment and are considered essential in modern dementia care.^5–7^

Increasingly more countries have developed national dementia care plans that provide guidelines for dementia care, and different care solutions are continuously tried out.^8^ However, research regarding which care approaches are used and what the outcomes are when met with resistance from home-dwelling PWDs^1,2,9^ is scarce. In a recent study from Belgium, home care nurses reported a prevalence rate of 52% involuntary treatments used within this patient group.^10^ Faced with a growing number of home-dwelling PWDs, we argue that knowledge of how to best provide basic care and how to prevent unnecessary use of coercion in PWD who resist care, is of key importance for those who deliver services.^1,2,8,11^

When PWDs resist care, it is a challenge to maintain voluntariness in the situation.^12–14^ Many home-dwelling PWDs want to take care of themselves at home for as long as possible and want to be treated with dignity, inclusion, and human warmth.^15,16^ Within existing research, approaches that are individualized, scalable, and flexible have therefore been highlighted as important to dementia care.^2,17^ Gastmans et al.^18^ introduced three key concepts as a foundational ethical framework for dementia care practice: vulnerability, care, and dignity. The philosophy of person-centered care emphasizes that PWDs need high-quality interpersonal care that meets fundamental and individual needs and stresses that the care relation implies recognition, respect, and trust.^6^ Trust-building interventions to reduce resistance and prevent involuntariness are one type of person-centered care approach for PWDs.^7,19,20^

### Background

Within a person-centered approach, the concepts of trust and trust-building can be understood at different levels. From an ontological perspective, trust may be understood as something fundamental that exists in humans and as a sovereign expression of life.^21,22^ However, within healthcare organizations, trust may often be reduced to a product, something that can be worked on.^22^ At the same time, trust is believed to be a fundamental asset when providing healthcare, and it is a prerequisite for the development of a functional care relationship between a health professional and a PWD.^23^ The rationale for emphasizing trust-building between health professionals and PWDs in the chapter 4A of the Norwegian patient and user rights law that regulates coercion is that healthcare is fundamentally voluntary. The aim for making trust-building interventions mandatory is to get in a position to secure needed healthcare in the least invasive manner, for PWDs who resist help and lack capacity to consent.^20^

The movement from the paternalistic approach to care practices where individualization, user participation, and voluntariness in care for PWDs is emphasized is widely and rightly integrated into the vocabulary of care guidelines and modern legal regulations for PWDs.^24–27^ Together with the implementation of a person-centered approach, it has enhanced dementia care practice.^6,28^ In later years, the concept of citizenship for PWDs has enhanced dementia care practices even more by adding a contextual, environmental, and political approach, for example, promoting age-friendly or dementia-friendly societies.^28–31^ At the same time, there are great challenges associated with how to assess and practice quality, safety, and autonomy in the care of home-dwelling PWDs who resist it.^10,13,32^

### Research aim

The aim of this study was to explore the use of trust- building interventions in home-dwelling PWDs resisting care, as described by health professionals in documents of decisions of forced treatment and care.

## Research design

Inspired by critical realism,^33-35^ a qualitative thematic analysis was performed on documents with texts describing trust-building interventions of care. A critical realist perspective on clinical practice emphasizes the importance of context and the multidimensional factors that may influence it, and it elucidates the complexity of situations.^33,35^ In our study, the perspective was found useful for exploring descriptions of ethically and clinically challenging situations in which trust-building interventions were initiated to prevent the use of coercion with home-dwelling PWDs who resisted care.

### Participants

The data set regarding trust-building interventions contained texts from 88 formal documents regarding decisions for forced treatment and care made for home-dwelling PWDs. The documents were written by health professionals in charge of and providing home-healthcare services to home-dwelling PWDs and were submitted for control to the County Governors Offices in Norway (CGO) in the period from 1 January 2015 to 31 December 2016. Altogether, the 88 decisions represented 352 pages, with a total of 30 pages describing trust-building interventions. These varied from a couple of lines to a little over a page for each document (for each PWD). By reading through the text of each document, an assessment was made of whether the patient’s lack of capacity to consent was caused by dementia.

### Data collection

The preparation for data collection was done in cooperation with one of the CGOs in Norway, where a pilot of the study was conducted. All 17 CGOs in Norway were invited to participate through a formal letter with information about the research project. Each CGO was required to extract relevant data from their own electronic case management systems. Case management actions that were performed and documented after the decision to provide forced treatment and care was submitted were not included in the study. A prepaid envelope with the return address on it was attached to the invitation letter. An e-mail reminder was sent 2 weeks later after the letters were posted. The principal researcher (Å.G.) and a case manager at one CGO were available for questions regarding the data-collection process. After the suggested submission date passed, the non-responding CGOs were contacted via telephone or e-mail. Eight CGO’s participated by anonymizing the data before sending them to the principal researcher. The principal researcher collected the data from one CGO herself with advance permission from The Regional Ethical Committee for Medical and Health Research Ethics number 2017/788. For the 2-year period from 2015 to 2016, there were gathered 116 documents.^36^ Eighty-eight of these contained information about use trust-building interventions to home-dwelling PWD. Most decisions were documented using a standardized form developed by the national health authorities in Norway to ensure that decisions for coercive healthcare are processed and documented according to law.^37,38^

### Research context

The standardized form requires a description of why the decision for coercion was needed and which trust-building interventions were applied and how, prior to the use of forced treatment and care.

**Figure 1.**
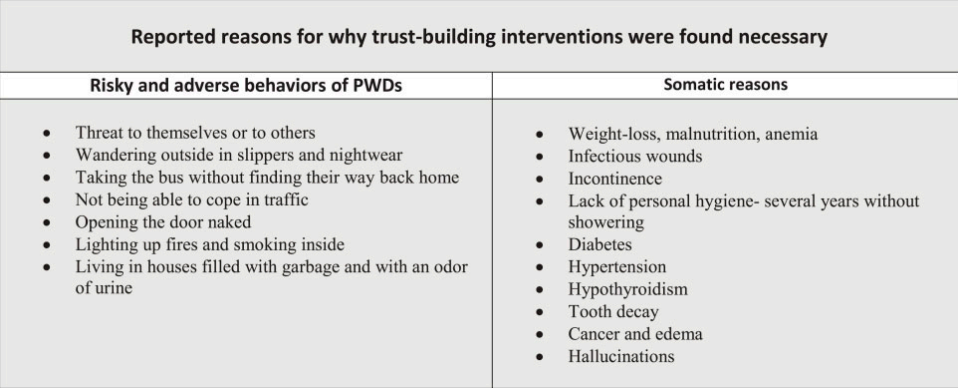
Characteristics of PWDs’ situations reported in 88 documents of forced treatment and care in Norway (2015–2016).

All the documents provided descriptions of PWDs with limited insight into their own situations and who could no longer take care of their own needs. Many were described as experiencing unsettlement, anxiety, and confusion. Severe somatic health needs were also reported as the reason for needed interventions (Figure 1).

#### Analysis

The thematic analysis used was inspired by template analysis as described by Brooks and his colleagues,^39,40^ which enables researchers to start with a priori themes identified in advance. Such themes may be temporal and redefined or removed if they do not prove to be useful for the analysis.^39^ We started with three a priori themes, defined as structural, relational, and individual trust-building interventions, to initiate the analysis of the data set. These a priori themes were seen as appropriate and useful categorizations of levels of interventions in this particular data set when considered against previous research on person-centered care, existing guidelines for legislation regarding forced treatment and care of PWDs, and a systems approach to care.^7,25,38,41^

#### Data analysis process

**Figure 2.**
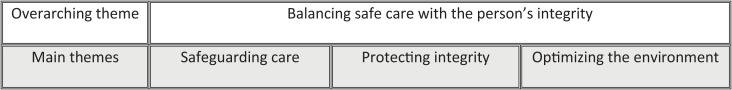
Final themes of trust-building interventions.

The data analysis process was performed in two phases. To obtain a general impression, the first author (Å.G.) read the whole data set of 88 decision documents, and all text concerning trust-building interventions was identified and extracted from the PDF files of the original documents and transcribed into a separate Word file. Then, three of the researchers/authors (Å.G., T.O., F.B.) conducted an initial analysis of the text. Preliminary coding using the three a priori themes of structural, relational, and individual trust-building interventions was carried out for 10 documents. Next, we applied this template to the whole data set individually and then together as a research team. Where coding diverged, we discussed until a consensus was reached. The whole research group (Å.G., T.O., H.A., F.B.) went through a new process of whether to keep the previous three a priori themes in the further analysis after a re-reading of several documents. In this process, we decided to remain open to new themes that could be identified. Then, all text concerning each theme was transferred into three separate Word documents to facilitate further analysis. The document number followed the text in the process to enable the possibility of going back to the original text. However, in accordance with the view of Brooks et al.,^39^ the a priori themes did not have a protected status. During the process of analysis, other themes, such as safeguarding care, were identified as being more fitting names for the data than the initial a priori themes. At this point, the categories of the a priori themes were therefore removed. The data were then coded into themes and subthemes, and finally, the themes were organized into meaningful clusters, and relations between them were identified. The analysis was an iterative process, and we moved from the context of the text to the individual parts of the text, allowing the parts to inform each other. An overview of the final themes, subthemes, and the overarching theme is provided in Figure 2.

#### Translation procedures

During translation, text quotes were transcribed verbatim and then modified to obtain equivalence in meanings and interpretations.^42^ All quotes used in the manuscript were numbered to make it possible to double-check their meaning. All names associated with the quotes have been changed to protect the identities of the patients.

### Ethical considerations and approvals

This document-based study did not involve direct contact with patients or healthcare professionals. The study was granted ethical approval from the Regional Committee for Medical and Health Research Ethics (REK) (reference number 2017/788). Moreover, it was presented to and received no objections from the Norwegian Centre for Research Data (NSD) (reference number 54897).

## Findings

We found that “balancing safe care with the person’s integrity” was an overarching theme that permeated the descriptions of trust-building interventions in the study (Figure 1). Three main themes were identified when the data were analyzed: *safeguarding care*, *protecting integrity*, and *optimizing the environment.*

In our data, the story about Anna provided us with important insight into how trust-building interventions were implemented to balance the safety and integrity of PWDs.

Our first contact with Anna was back in 2004 when we assisted her with domestic care due to her reduced mobility. Gradually, Anna’s care needs increased, and she needed assistance with all personal hygiene. In 2007, she was diagnosed with dementia. By this time, she would resist strongly when we tried to help her. However, it was not until 2013 that she was included on our dementia team list. During the last two years, Anna’s physical and mental health deteriorated severely. It was a dilemma that her unmet needs seemed to increase proportionally with her anxiousness. We needed to carefully balance being close and being distant. Eventually, her situation came to a point where we considered it unsafe and no longer a dignified situation for Anna to remain living at home. We admitted Anna to a nursing home against her will in December of 2015.

### Balancing safe care with the person’s integrity

We found that an overarching theme in the health professionals’ descriptions of their work with trust-building interventions was the balance between establishing trust and using coercion to provide needed healthcare. They attempted to respect the preferences of the PWD, which often included little-wanted intervention and also to provide enough safety for the person. Safety was referred to as both an objective term of “patient safety” and as a subjective term for the PWD’s “feeling” of safety.

Common limitations to be able to remain living at home seemed to be a sudden decrease in support from family members due to illness, exhaustion, or where there was threatening and violent behavior from the PWD. Also, when an increased number of home visits was no longer enough to provide needed care, this was a turning point for the continuance of home healthcare like in the story of Anna where it came to the point when she could no longer be left alone without supervision.

#### Safeguarding care

One of the main themes was concerned with safeguarding care for the PWD. Adaptation of structures and content of care were assumed to be important interventions for a PWD who resisted help, with the intention of being able to approach the PWD.

As far as possible, we try to have regular and familiar staffing to establish safety and predictability for the patient. For some time, we have also had temporary staff members follow up and reassure Eirik in the ward. This has not been effective so far. We have also made an intervention plan to ensure that staff do tasks the same way so that the situations become as predictable as possible.

In the analysis, we also found that adjusting the content of care by selecting staff members with experience and competence in dementia care was common. In a few documents, care plans for how to approach the PWD were referred to. Such plans could imply everything from serving coffee and cake before offering to help with personal hygiene (and getting into a position to care) to detailed descriptions of how to approach the PWD.

When a PWD refused home healthcare, teamwork and multidisciplinary collaboration was often applied to safeguard thorough assessment. The general practitioner and dementia teams would do home visits to try to assess the PWD, and professionals and family members would often collaborate in the process of gaining entry to examine or provide healthcare to the PWD needing help.

Home health care services have over a longer period tried to motivate and speak with him to inform him about the importance of receiving care within an institution. There has been multidisciplinary collaboration between home nursing, a substance-abuse consultant, the physician, and the dementia coordinator, where all of them have tried to motivate Lars to accept a stay at the nursing home. This is to avoid the use of coercion. Lars becomes irritated and clearly states that he does not want to move.

As equally important to a competently professional care team, the level and continuity of care was illuminated. Like Lars from the quote above, many of the PWDs needed a higher level of care, often a nursing home, which they did not want. In the process of motivating the person to accept a higher level of care, staff members sometimes referred the patient to outpatient clinics or short-term stays at nursing homes to see how they reacted or adapted. Frequently, the patients refused to go, or they discharged themselves shortly after admission. And even though family members needed respite, enduring forced placement was difficult for them. Several documents reported that family members would end up taking the PWD back home.

Overall, health professionals stated that they strived to provide a structure of care surrounding the patient that would protect them from harm while enabling them to continue to live at home as long as possible or reducing interference with their free will. When the PWD’s health declined, or in cases where they did not want to move, home healthcarers tried to safeguard care through increased intensity with more frequent home visits, sometimes up to four to six times a day.

To be able to get in a position to help and create trust, the health professionals emphasized having a careful and calm approach to the situation. Especially in situations involving personal hygiene, maintaining dignity was repeatedly described as fundamental. Creating a good atmosphere was highlighted. Also, careful hand-leading and eye contact in the situation was highlighted. Not acting in a desecrating manner was emphasized, and establishing a relationship of trust was fundamental.

Also, foreseeing irritability and uneasiness due to natural functions, such as having to use the toilet, was an important part of trust-building. It was important to avoid an escalated situation where coercion might be necessary. In challenging situations where resistance was expected, the care team would sometimes take on “different roles,” with one nurse communicating and emotionally supporting the patient while another nurse was doing the practical work, such as undressing and washing. Clear communication, using few or maybe just single words to explain what was going to happen, was stressed. If one nurse was pushed away, the other would try to take over. Sometimes, they would try again later. An awareness and use of such roles were highlighted.

A careful approach could imply planning for the PWD to have few and familiar professionals to relate to. Also, continuity of care over time was believed to be important for establishing a feeling of predictability and safety.

These are nurses that the patient is familiar with and that he is safe with. The nurses have participated as support, have comforted the patient, and have tried to create safety during the treatment.

Family members were often described as being used like trusted allies. They could be physically present during interventions because their presence would calm the situation. Collaboration with family members also took place outside the actual care situation as motivators or persuaders to encourage the PWD to accept help.

#### Protecting integrity

The particularity of the individual case was the focus, and when the PWD could not actively participate or verbally express their preferences, family members, if existent, were asked to express their opinions. Health professionals emphasized the individual approach and tried to comply with the PWD’s habits and preferences as far as possible.

The findings identified that many PWDs wanted minimal interference in their daily routines, and it was a continuous dilemma that the increased presence of health professionals could lead to increased suspicion and resistiveness on the part of the PWDs. Respecting the patient’s choice was regularly presented as the preferred approach. Thus, it was necessary to work with the PWD to get in a position to help; however, it was often not easy to intervene with trust-building interventions.

Home health care now visits in the morning, evening, and during the night, if needed. Her general practitioner has visited on various occasions to follow up on her health situation. Because she periodically becomes very unsettled with increased supervision, this has implied that home health care has needed to balance care between being close and being distant.

The health professionals would try to tailor interventions so that the PWD could remain living at home for as long as possible, but sometimes it seemed implicit in the descriptions in the documents that interventions were not seen as possible to conduct. The following situation indicates that it was considered necessary to forcefully admit the PWD to a nursing home.

She lives at home and has been taken care of by her husband, who has provided assistance with personal hygiene, cooking, and other tasks. He died the first of January.…The patient does not have the capacity to take care of herself at home. She does not understand when she needs food, that the stove needs to be turned off, that she needs to go to bed, etc. She has said that she does not want to leave her home, and she doesn’t want help; she wants to manage on her own…Her niece explains that the patient moves in and out of grieving for her husband. One moment she looks for him, the next she grieves over him.

The constant weighing between being close and being distant was not without risk for patient safety. In some documents, there were descriptions of PWDs who would leave their home for long periods of time and where there was uncertainty about their whereabouts or well-being. Sometimes, the PWD would be brought home by the police, and the health professionals in charge of care were aware that if the PWD left home without supervision, it could be attached to a great deal of risk. To try to protect them, the health professionals therefore increased the frequency of home visits and thus the possibility to supervise the person. This was done to ensure that the patient was not left alone for longer periods of time, and if the PWD had family, supervision was often done in collaboration with them.

In this balance of being close enough to safeguard the PWD’s well-being while allowing for them to have personal space and freedom, remaining flexible and adjusting to the person’s daily routines while trying to provide a minimum of services, which was often only supervision, were described as important.

#### Optimizing the environment

The health professionals highlighted the importance of a physical environment inside and outside that accorded with the principles of dementia care. Especially in care homes with small units of seven to eight persons, private rooms and bathrooms, personal belongings, and common areas adapted for PWDs, the physical environment was illuminated as beneficial for well-being. Furthermore, individual adjustments were also highlighted, such as using black toilet seats to make them more visible (and recognizable) and hanging up signs with “toilet” for a PWD who could still read. Also, various social activities, such as singing and the possibility of religious worship, were offered to PWDs to stimulate them, bring meaning to daily life, and maintain their tranquility.

The resident lives in a department with seven other residents. Here, she has her own room and bath, with personal belongings, and a shared kitchen and living room. Additionally, she has access to a fenced therapeutic garden. There is usually continuity in the department staff to create safety and predictability for the patients. The patient is offered various activities, such as group singing, devotional time, walks, and so on.

As we can see from the above quote, the outdoor environment was emphasized, and some also described the therapeutic use of the gardens to stimulate senses. In general, easy access to adapted outside areas that allowed for walking alone, but also prevented the PWD from leaving unsupervised, were highlighted as fortunate. For PWDs who could walk safely alone, GPS was considered a less invasive option. The nurses would also take walks and go shopping together with the PWD to prevent uneasiness.

However, for PWDs living in their original homes, the outdoor physical environment was less emphasized. Individual adjustments were also made inside private houses, but these were smaller adaptations, such as removing carpets to prevent falls and installing new kitchen facilities that would be automatically turned off, safer heating equipment, and safety sensors and alarms.

### Strengths and limitations of the study

The data analyzed were collected from pre-existing documents from 2015 and 2016. Although some health professionals were thorough in their descriptions of the trust-building interventions applied, many descriptions were limited. Most of the documents described the severity of the PWD’s situation more thoroughly than the trust-building interventions. The reason for this may be that further interventions were considered purposeless. The age of the data could be a limitation; however, legislation and structures surrounding home healthcare have not changed considerably since the data collection.

The strengths of this study are the nuances and variations of the descriptions and examples of trust-building interventions provided in the data. Moreover, the study increases understanding of the work of home-healthcare professionals when meeting resistance from home-dwelling PWDs. The findings add unique knowledge about PWDs who resist care.

## Discussion

A fundamental question in this study was whether trust-building interventions were aimed at creating trust or were primarily used as ways to overcome resistance and get in a position to help safeguard care. The findings identified that health professionals utilized various strategies based on different reasons out of concern for the PWD when met with resistance. At the same time as the findings indicated an awareness of that trust needed to be established for its own sake, to strengthen the PWD’s feeling of safety and to protect the person’s integrity, trust-building was often aimed at overcoming resistance to secure the PWD’s safety.

Our data demonstrate that clinical practice in home-health dementia care is layered and complex. It is influenced by physical and organizational structures, the competence of the health professionals, by family, and it is dependent on the preferences of the PWD. From a critical realist perspective, this article argues the importance of being aware of structures and practices that may influence various outcomes of dementia care.^33-35^

We found that safety and the safeguarding of care were major concerns of health professionals when the PWD resisted care, and this is supported by previous research.^10,43–45^ The examples from our data were related to challenging situations of care, and many of the situations were followed by forced placement in a nursing home. The challenging characteristics of the situations described were probably due to the starting point of care at which the PWD had already resisted care. The health professionals described that they provided flexible and scaled services of care to get into a position to help and to keep the PWD safe. The importance of a flexible approach to dementia care has been previously highlighted in research.^2,17,46^ The findings in our study are not necessarily representative of care to all home-dwelling PWDs, as they were concerned with situations of resistance. Nevertheless, the descriptions of interventions provided were recognizable from previous research.^10,47^ In our study, the main reasons that trust-building interventions were implemented to safeguard care for the home-dwelling PWDs were related to descriptions of poor hygiene, inadequate nutrition, risk of falling, and need for supervision. These risks have also been identified in previous research.^10,48,49^

The findings suggested that multidisciplinary approaches were common, which allows for a competent assessment of the situation. The approach was characterized by a combination of competence and knowledge in the care team, by adapting the content of care, and by increasing the intensity and levels of care. In line with previous research, we also found that the type and extension of the trust-building interventions implemented were influenced by context, that is, the preferences of the PWD and family members, other health professionals, and the limitations of possible supervision within structures of home healthcare.^50,51^ Such a multidisciplinary approach is in line with existing legal guidelines, where multidisciplinary collaborations are mandatory for very intrusive interventions of forced treatment and care.^20^ Family members were also involved in the interventions in different ways. They would act as caregivers for the PWD, either living with them or living in the same house but in separate “apartments.” A few received allowances for the care of family members. Many agreed that health professionals could intervene with coercion due to a concern for unmet needs and the security aspect. Family members would also convey the PWD’s voice, providing information about their past habits and preferences if the person could no longer do so.

Although integrated and coordinated healthcare services has been recognized as especially crucial for PWDs, previous research has found that collaboration between general practitioners and home healthcare is neither regular nor systematic.^52^ This raises the question of whether a practice of multidisciplinary teamwork, and especially collaboration with the PWD’s general practitioner, is enabled within the existing context of home healthcare.^52,53^ As previously mentioned, our findings were concerned with relatively few but challenging situations, and some documents described years of trust-building interventions from home healthcare. It is uncertain whether a systematic multidisciplinary approach at an earlier stage could have been beneficial for the PWDs.

Health professionals were not only occupied with practicing safe care; they also dedicated major attention to individual adaptation of care to protect the integrity and the possibility of self-determination by the PWDs and went to great lengths to meet the PWDs’ needs and preferences. The significance of home and the ability to remain living there as long as possible is well known from previous research,^54^ and this was also described as one of the main reasons for the adaptation and flexibility of care in our study. Many PWDs expressed that they wanted to live in their private original houses. The individual approach to the PWD’s situation in our findings is in line with the recommendations of a Lancet commission for individualizing dementia care.^17^ Through protecting the integrity of the PWD by respecting their wishes, the health professionals attempted to get into a position to help by establishing a relationship and feeling of trust. Key approaches were to proceed carefully, utilize dementia-friendly communication strategies, provide flexibility, and respect the PWD’s preferences. The importance of a careful and dementia-friendly approach accords with a fundamental understanding of the phenomenon of trust and what allows trust to occur. How to “set the tone when meeting the other” like Løgstrup says it, is crucial for further communication.^21,55^ In their individual approach, the health professionals in our study described how they needed to balance closeness with distance to enable the possibility of at least a minimum level of supervision. The necessary flexibility of when and how to do home visits was especially illuminated. The rationale for the strong focus on protecting integrity may also be recognized in guidelines for care and legislation in the person-centered care philosophy. Here, the emphasis on self-determination, individual choice, and the *person* are presented as fundamental tenets.^7,20,25,56,57^ Gastmans et al.^18^ provided a critique and counter-approach to a unilateral understanding of self-determination in PWDs with the introduction of the ethical framework for dementia care practice that also included the concept of vulnerability. In PWDs, a balanced assessment of dignity, care, and vulnerability is necessary to provide quality and safe care.^18^ The increased expectation of user participation in healthcare adds complexity to situations of resistance in that it implies a need for great effort, responsibility, and knowledge from both the health professional and the individual PWD, which they may or may not manage (or want) to fulfill.^26^ We support the dilemma presented by Jacobsen and Sundsbø,^58^ that home-dwelling PWDs have a need not only for predictability, safety, and security but also for maintaining integrity through self-determination, a rich social life, activity, and fellowship.^58^ Therefore, appropriate assessments of how to weigh respect for a PWD’s decision to resist care against the need for care are fundamental and complex but of great importance.

Another important finding illuminating the application of trust-building for home-dwelling PWDs was optimization of the environment. In our findings, the design and modification of the physical architecture and the inclusion in social activities were described as important interventions to maintain as much self-dependency as possible for PWDs living in care homes. The health professionals highlighted that the care homes where the PWDs lived were adapted for PWDs. They pointed to the choice of colors, how many people lived and interacted together, and access to outside areas for people who were restless and liked to walk. This emphasis on the importance of an external environment to maximize the performance potential of PWDs is in line with recommendations in previous research and guidelines for age-friendly construction.^30,59–63^

At the same time, for PWDs who lived in their private homes, the optimization of the environment was less emphasized in the findings. This was not surprising, as adaptations in and outside of private homes could implicate strong collaboration between the PWD, family, and the immediate neighborhood, something that could prove difficult and that might be considered outside the area of responsibility of the home-healthcare services. Still, in consideration of the increase in the number of PWDs who live at home, it would be interesting to examine whether a dementia-friendly environment could influence and ease dementia care for home-dwelling PWDs who resist help, especially dementia-friendly initiatives focusing on outdoor facilities or inclusion in society. A recent governmental review points to growing research on the potentials, user preferences, and challenges of planning for age-friendly societies.^58^ This review also presents promising examples of local societies adapting the environment through the colocation of age-friendly apartment buildings and nursing homes, home-healthcare offices, stores, movie theaters, cultural arenas, localization of sports and youth activities, and parking.^58^ There is still more research needed on the possible impact of dementia-friendly societies, including universal designs of buildings and transportation systems, educating staff in shops about dementia, enhancing neighborhood relations, accessing normal activities (including the challenges of seasonal variations for mobility), and inclusion in an increasingly digitalized society, on PWDs who resist help.^59^ Ward et al.^63^ have presented valuable insights on the topic, showing that neighborhoods are a primary locus either to enable or constrain the key features of social health for people living with dementia (p. 877).^63^ Their research explored the “person-in-environment” of PWDs living in private homes and examined how they experienced their lived space.^63^ Important implications from their research could be that, in assessing the needs of home-dwelling PWDs, mobile assessment and walking interviews could give valuable information to health professionals about the PWDs’ needs and how they function in their closest neighborhood.^64,65^ There seems to be a knowledge gap regarding whether environmental initiatives may also benefit home-dwelling PWDs who are not easily cared for and who resist care. Bridging this gap would be an important contribution to future dementia care research.

In this final section, we turn our attention to the practical ethical discourse on ethics, care, and aging,^3,18,62^ as an under-communicated dilemma in professional judgment of the balance of how long to continue with trust-building interventions and how to intervene when a PWD resists care. Tranvåg et al.^62^ has highlighted the same dilemma related to balancing individual choices among persons no longer able to make sound decisions, against the duty of making choices on behalf of the person. In some situations, the mechanisms of person-oriented care or trust-building interventions may not be successful in creating voluntariness or acceptance from the PWD. This may have many explanations, ranging from non-competent care, lack of structures to support care, lack of infrastructure in society to support the PWD in their daily life, to the severity of the dementia itself. Nonetheless, these situations need to be acknowledged as existing and dealt with in the short and long term to enable improvement in care for home-dwelling PWDs who resist help.

This study provides new insight into the field of tension and ethical blindspots that exist when using trust-building interventions to prevent coercion with home-dwelling PWDs. From previous research, we know that it is common for health professionals to encounter PWDs who resist care, and that they are required to find ways to provide care for them.^10,12,62^ The findings from our study provide examples of complex situations where it was almost impossible to get into a position to help and where respect for the PWD’s preferences and integrity seemed difficult to balance against patient safety. This study describes the most challenging situations in home healthcare, when health professionals assess that the need to use forced treatment and care is the only remaining option. However, the toolkit they use in these situations do not seem to differ from what is used in standard dementia care. They use the same trust-building interventions for the most challenging situations with resistance as they do in less complicated situations.

The practical ethical analysis of caregiving for home-dwelling PWD who resists care needs to be developed and challenged. The level of competence in health professionals’ interventions and decision-making practices, as well as the structures and surrounding environment when encountering PWDs who resist care, is important in this context and may be crucial to the outcomes of the healthcare service provided.^18,51^ The decision of how and when to intervene in a situation of resistance puts a heavy load of responsibility and demand for competence on the individual health professional in the situation. In our study, a multidisciplinary approach was initiated in the most difficult situations and included the general practitioner, dementia specialists, and family, in addition to home-healthcare staff. However, this approach requires that the advantage of collaboration is understood by the individual health professional, enabled in the structure of home healthcare and that requests are welcomed by the general practitioners.

## Conclusions and the need for future research

Health professionals balanced on a thin line between care and integrity when met with resistance from PWD. However, the trust-building interventions used in the most challenging situations did not differ from the interventions used in general in dementia care. We argue that the operationalization of policy, ethics, and legislation, and the practical responses to care for home-dwelling PWD need to be explored further to enhance both somatic care needs and patient rights for this patient group.

Two knowledge gaps were identified: how to perform appropriate assessments of situations of home-dwelling PWDs when met with resistance to care, and whether environmental initiatives may also benefit home-dwelling PWDs who are not easily cared for.
